# Optimizing polyamide thin-film composite desalination membranes: simple and performance-effective modification by zwitterionic monohydroxyl monomer

**DOI:** 10.55730/1300-0527.3740

**Published:** 2025-04-25

**Authors:** Selda ERKOÇ İLTER

**Affiliations:** Integrated Manufacturing Technologies Research and Application Center & Composite Technologies Center of Excellence, Sabancı University, İstanbul, Turkiye

**Keywords:** Desalination, reverse osmosis, zwitterionic hydroxyl monomer, thin-film composite, high flux, polyamide membrane

## Abstract

A synthesized zwitterionic monohydroxyl monomer (ZHM) was used as an additive to incorporate zwitterionic sulfobetaine groups into the polyamide (PA) active layers of thin-film composite membranes. Incorporation of ZHM into the PA active layers was achieved through interfacial polymerization, involving the introduction of ZHM and m-phenylenediamine (MPD) into the aqueous phase, and the addition of trimesoyl chloride (TMC) to the hexane phase. The surfaces of the resulting reverse osmosis (RO) membranes were subjected to characterization through water contact angle (WCA) and field emission scanning electron microscopy (FESEM) analyses. The successful incorporation of ZHM into the active PA layers was confirmed by X-ray photoelectron spectroscopy (XPS) and Fourier transform infrared spectroscopy (FTIR) analysis. To assess the flux and salt rejection performance of the fabricated membranes, aqueous solutions containing 2000 ppm NaCl or MgSO_4_ were filtered in a dead-end filtration system under a pressure of 15 bar. Compared to the control membrane (ZHM-0), the ZHM-modified membranes had significantly enhanced flux without compromising salt retention. In the NaCl solution filtration, the modified membrane (ZHM-1) increased in flux from 6.8 to 9.1 L/m^2^h while having similar salt rejection (approximately 91%) in comparison to the control membrane (ZHM-0). In the MgSO_4_ solution filtration, the modified membranes increased in flux from 6.7 to 9.5 L/m^2^h, while maintaining a consistent salt rejection rate of 100%, mirroring that of the control membrane.

## 1. Introduction

The advancement of innovative desalination membrane materials is a crucial area of research, with the aim of developing membranes that offer improved performance and enhanced energy efficiency [1–3]. Over the years, there have been notable developments in membrane materials, particularly for reverse osmosis (RO) desalination [1,4–8]. Cellulose acetate-based membranes were the earliest materials used for RO desalination membranes, dating back to the 1960s [9,10]. They typically consist of a selective, ultrathin surface film over a microporous thicker support layer to provide mechanical strength. These membranes have integrally skinned asymmetric structures produced through the phase inversion method. Following cellulose acetate-based membranes, thin-film composite (TFC) membranes were a significant advancement [11–13]. In TFC membranes, the active surface layer is created via interfacial polymerization of reactants on a support layer that offers mechanical stability^1^. Typically, the active layer consists of aromatic polyamide (PA), synthesized by the interfacial polymerization of widely used monomers such as trimesoyl chloride (TMC) and m-phenylenediamine (MPD). This construction allows for a more efficient separation process and better performance compared to earlier generations of RO membranes. Nevertheless, it is essential to achieve an improvement in water flux efficiency without compromising salt retention during desalination operations.

Zwitterionic structures have distinct and advantageous properties for membrane technology [14–22]. A zwitterion is a molecule distinguished by the presence of both positive and negative charges on separate functional groups, such as carboxybetaine, phosphobetaine, or sulfobetaine within the same structure. These functional groups can be incorporated onto membrane surfaces through various methods such as coating, grafting, or deposition [21,23–28], and within the bulk of the membrane through some chemical reactions or physical blending [29–31]. Over the years, a diverse array of materials has been utilized for both surface and bulk modification of membranes in various applications. This includes the incorporation of different zwitterionic polymers, monomers, and hybrid nanomaterials to enhance membrane performance [24,30,32–34]. Zwitterionic structures can create electrostatically induced hydrophilic pathways within the membrane matrix. These pathways facilitate the movement of water molecules through the membrane, leading to enhanced water permeability without compromising selectivity. The balanced distribution of positive and negative charges induces hydration of the surface, making it more attractive to water molecules [35,36]. In addition, zwitterionic structures on the membrane surface can resist fouling by biomolecules and other contaminants due to their hydrophilic nature [37,38]. An electrostatically induced hydration layer generated by zwitterionic structures prevents the attachment of foulants onto the membrane surface, thus maintaining membrane performance over time.

Zwitterionic or charged functional groups have been integrated into TFC membranes through 2 main approaches. One method involves incorporating charged functional monomers during the interfacial polymerization process, allowing these groups to be directly embedded within the PA selective layer [39,40]. Alternatively, zwitterionic functionality has been introduced postsynthetically by modifying the membrane surface through chemical reactions or coating techniques [23,28,41–44]. For example, Li et al. [45] developed a novel nanofiltration membrane (NFM) by designing an amine monomer, 4-(piperazin-1-yl)benzene-1,3-diamine (PMPD), which combines piperazine (PIP) and MPD units in a single molecule. This monomer was polymerized with TMC to form the base membrane that was later modified with 3-bromopropionic acid (3-BPA) to introduce zwitterionic antifouling properties. The resulting PMPD-PA membrane had high Na_2_SO_4_ rejection (98.4%) and low NaCl rejection (24.1%), making it a promising selective NFM. It also performed well in antifouling tests with BSA and SA. In our previous studies, we synthesized a zwitterionic functional trimethoxysilane compound and used it to modify PA-TFC RO membranes through interfacial polymerization [34] and surface coating [28]. Modification via interfacial polymerization led to enhanced water flux, increasing from 25 to 33 L/m^2^h in seawater desalination without compromising salt rejection. Membranes modified by surface coating had improved salt retention, albeit with a slight decrease in water flux during the filtration of a 2000 ppm NaCl solution. Le et al. [46] prepared NFMs through interfacial polymerization using a zwitterionic amine monomer. In their study, they utilized a zwitterionic functional triamine monomer in conjunction with MPD in the aqueous phase, along with TMC in the organic phase. The resulting membranes showed enhanced water flux while preserving high retention rates for dyes with molecular weights ranging from 637 to 1673 g/mol. In addition, the development of a membrane with a smoother and more hydrophilic surface provided enhanced fouling resistance, as evidenced by minimal protein adsorption and little decrease in permeance during filtration processes. Ma et al. [47] introduced a zwitterionic diamine monomer N-aminoethyl piperazine propane sulfonate (AEPPS) into the PA layer of TFC RO membranes via interfacial polymerization to produce zwitterionic TFC RO membranes (TFCMZs). Consequently, the modified RO membranes had improved separation efficiency when operating under brackish water conditions (2000 ppm NaCl solution filtration, 15 bar, and 25 °C). An et al. [48] incorporated a zwitterionic diamine monomer (AEPPS) with PIP during interfacial polymerization with TMC to fabricate TFC NFMs. This enhanced the separation performance and antifouling properties of the membranes. When tested with a 1000 ppm K_2_SO_4_ solution at 25 °C and 0.6 MPa, water flux increased from 23.4 to 43.1 L/m^2^h as the AEPPS content increased from 0 to 3.2 mol%, while K_2_SO_4_ rejection remained stable at approximately 97%. Additionally, the zwitterionic-modified NFMs effectively resisted bacterial adhesion and protein fouling. Incorporating polyamine or hydroxy functionalized zwitterionic aliphatic monomers in interfacial polymerization enhances water permeability and imparts antifouling properties to the resulting membranes [17,49]. However, it is essential to note that their polyfunctionality may compromise selectivity by creating aliphatic voids within the membrane matrix [46]. To overcome this, integrating aliphatic zwitterionic structures directly into the primary aromatic framework without disrupting the aromatic continuity can enhance the overall hydrophilicity of the membrane while preserving its intrinsic structure. This approach ensures improved permeability without sacrificing selectivity.

**Figure 1:**
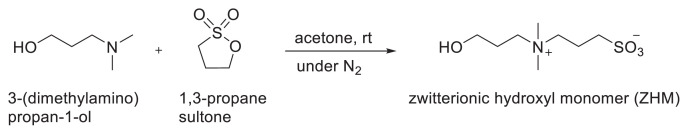
Synthesis of the ZHM.

**Figure 2:**
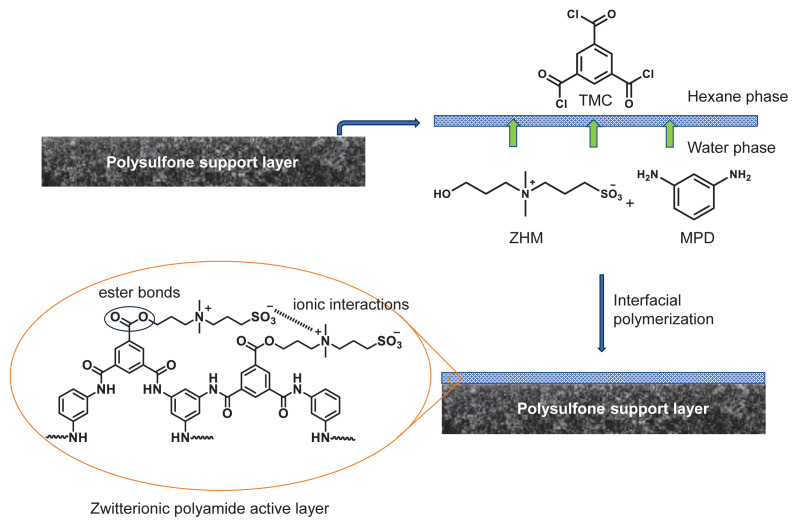
Formation of zwitterionic PA top (active) layer by an interfacial polymerization of ZHM and MPD in the aqueous phase with TMC in the hexane phase.

In the current study, the aromatic PA active layer in PA-TFC RO desalination membranes were modified using a zwitterionic monohydroxyl monomer (ZHM) for the first time. With its facile synthesis and seamless integration into PA-TFC membrane fabrication, the ZHM additive is efficient and practical, thereby advancing desalination technology. The ZHM was synthesized through the reaction of *N*,*N*-dimethylpropanolamine with 1,3-propane sultone (Figure 1). Integration of ZHM into the PA active layer was achieved through interfacial polymerization. During this process, ZHM was added to the aqueous phase along with MPD, while TMC was added to the hexane phase. A schematic representation of the formation process of the zwitterionic PA active layer via interfacial polymerization is depicted in Figure 2. ZHM was integrated into the PA active layer by forming chemical ester bonds through condensation reactions between the hydroxyl (−OH) group of ZHM and the acid chloride groups of TMC (Figure 2). Five different zwitterionic-modified PA active layered TFC membranes were prepared by adding varying amounts of ZHM to the aqueous solutions. The performance of the zwitterionically modified PA membranes was evaluated against a control PA membrane prepared without ZHM. The membranes were chemically and morphologically characterized to verify the integration of ZHM into the PA active layers.

## 2. Materials and methods

### 2.1. Materials

The following chemicals were obtained from Sigma-Aldrich: (+)-10-Camphor sulfonic acid (CSA) (98%), sodium dodecyl sulfate (SDS), 3-(Dimethylamino)propan-1-ol (99%), 1,3-propane sultone (≥99%), m-phenylenediamine (MPD) (99%), 1,3,5-benzene tricarboxylicacid chloride (TMC) (98%), triethylamine (TEA) (99.5%), acetone (≥99.5 %), hexane, sodium chloride (NaCl, ≥99.0%), and magnesium sulfate (MgSO_4_, ≥99.5%). Prior to use, acetone and hexane were dried with calcium hydride and then distilled. Commercial ultrafiltration membranes served as support layers to produce TFC membranes, specifically the polysulfone JQM-PS-500 membrane (average pore size 0.05 μm, JiaQuan, Guangdong, China). All stirring bars and glassware underwent overnight drying in an oven at 120 °C and were purged with nitrogen gas prior to use. Deionized (DI) water from a Merck Millipore Direct-Q 3UV ultrapure water system was used for preparing aqueous phases, conducting filtration experiments, and performing analyses.

### 2.2. Synthesis of ZHM

The preparation of the ZHM followed the reaction depicted in Figure 1. The empty reaction vial equipped with a magnetic stirrer was sealed with a rubber septum and purged with nitrogen gas for 30 min. Subsequently, 20.6 g of 3-dimethylamino-1-propanol (0.2 mol, 23.6 mL), 26.8 g of 1,3-propane sultone (0.22 mol, 19.2 mL), and 100 mL of dry acetone were added to the reaction vial using a syringe under a nitrogen atmosphere. The mixture was then stirred at room temperature under a nitrogen atmosphere for 6 h. At the conclusion of the reaction, the white solid precipitate was washed 3 times with anhydrous acetone to remove unreacted reagent chemicals. The resulting pure product was subsequently dried in a vacuum oven at 30 °C overnight and stored in a vacuum desiccator. The structure of the compound was confirmed through ^1^H and ^13^C NMR analyses. The characteristic peaks were indicative of the purity of the compound (Figures S1 and S2). ^1^H NMR (CD_3_OD): 1.98 (m, 2H, −C*H*_2_CH_2_OH), 2.21 (m, 2H, −C*H*_2_CH_2_SO_3_), 2.88 (t, 2H, −C*H*_2_SO_3_), 3.11 (s, 6H, ^+^N(C*H*_3_)_2_, 3.44 (t, 2H, ^+^NC*H*_2_CH_2_CH_2_SO_3_), 3.53 (t, 2H, ^+^NC*H*_2_CH_2_CH_2_OH), 3.66 (t, 2H, −C*H*_2_OH) ppm. ^13^C NMR(CD_3_OD): 14.6 (−*C*H_2_CH_2_SO_3_), 22.0 (−*C*H_2_CH_2_OH), 46.4 (−*C*H_2_SO_3_), 54.3 (−*C*H_2_OH), 58.3 (^+^N*C*H_2_CH_2_CH_2_OH), 58.6 (^+^N*C*H_2_CH_2_CH_2_SO_3_) ppm.

### 2.3. Preparation of membranes

The surface of the support layer polysulfone ultrafiltration membrane was exposed to a water solution including 2% MPD, 1% TEA, 1% CSA, and 1.0% SDS (w/w). In the modified membranes, 0.1%, 0.2%, 0.5%, 1%, or 2% ZHM (w/w) was added to the aqueous solutions, and the resulting membranes were labeled as ZHM-0.1, ZHM-0.2, ZHM-0.5, ZHM-1, and ZHM-2, respectively. A control PA-TFC membrane was prepared without ZHM and labeled as ZHM-0. The weight percentages of components in the water and hexane phases used in interfacial polymerizations are provided in Table 1. The aqueous solution was left on the surface for 5 min to allow the surface of the support membrane to absorb the aqueous phase. Subsequently, the aqueous solution was removed from the surface, and any excess was removed with a rubber roller. After airdrying the membrane surface for 2 min, a hexane solution including 0.1% TMC was poured onto the surface of the membrane. Interfacial polymerization between the hexane and water phases was allowed to occur on the surface for 2 min. Following this, the hexane phase was removed from the surface. To enhance the crosslink ratio of the aromatic PA layer formed on the surface of the membrane, the membranes were cured in an oven at 70 °C for 10 min. Three sets of membranes were manufactured for each composition. These membranes were then preserved in distilled water until they were utilized in the dead-end stirred cell filtration system.

### 2.4. Characterizations

#### 2.4.1. NMR spectroscopy

^1^H NMR and ^13^C NMR analyses of the ZHM were conducted using a Varian Unity Inova 500 MHz spectrometer (Agilent, Santa Clara, CA, USA). Measurements were conducted at ambient temperature using a ^1^H-^19^F (^15^N-^31^P) 5 mm PFG switchable probe to record spectra. The ^1^H and ^13^C NMR spectra are presented in Figure S1 and Figure S2, respectively, with relevant annotations highlighting the chemical structure of ZHM.

#### 2.4.2. Fourier transform infrared spectroscopy

Fourier transform infrared (FTIR) spectra were utilized to confirm the structural composition of the active layer polymers. The spectra were recorded in absorbance measurement mode within the range of 4000–400 cm^−1^ at ambient temperature. A PerkinElmer 100 FTIR spectrometer (Waltham, MA, USA) with a resolution capacity of 4 cm^−1^ was used for data acquisition. A baseline spectrum was recorded prior to each sample to minimize atmospheric and instrumental interference.

#### 2.4.3. X-ray photoelectron spectroscopy

X-ray photoelectron spectroscopy (XPS) analyses were used to characterize the chemical structure and elemental content of the membrane surfaces. The Thermo Fisher Scientific K-Alpha spectrometer (Waltham, MA, USA) outfitted with an aluminum anode (Kα = 1468.3 eV) positioned at an electron emission angle of 90° was utilized for these analyses. Spectra were acquired using Advantage version 5.9 software.

#### 2.4.4. Field emission scanning electron microscopy

Field emission scanning electron microscopy (FESEM) analyses were conducted on the active layers of the membranes using the Thermo Fisher Scientifıc Apreo 2 S LoVac system. Before analysis, the samples were vacuum dried overnight and then coated with a gold-palladium (Au-Pd) alloy.

#### 2.4.5. Water contact angle

The hydrophilicity of the membrane surfaces was assessed by determining the water contact angles (WCAs) utilizing a KSV Attension T200 Theta contact angle measurement device (Biolin Scientific, Gothenburg, Sweden). At 25 °C, a stainless-steel syringe needle was used to place a droplet of DI water onto the dry membrane surface. Five contact angle readings were taken at 1-s intervals at 3 separate locations on each membrane, and the results were averaged to obtain the final value.

### 2.5. Membrane desalination performance

The desalination performance of the membranes was assessed using a Sterlitech HP4750 (Auburn, WA, USA) dead-end cell filtration setup equipped with a magnetic stirrer, with an efficient membrane region of 14.6 cm^2^. Desalination efficiency tests were carried out by passing a 2000 ppm NaCl or MgSO_4_ solution through the membranes. The experiments were performed under controlled conditions: a stirring rate of 300 rpm, at room temperature, and an operating pressure of 15 bar. After a 60 min stabilization period, salt retention and permeating flux performance of the membranes were determined. The permeate flux, denoted as *J* (L/m^2^h), was determined using Equation 1, where *ΔV* represents the volume of permeate collected, *A* represents the effective membrane surface area, and *Δt* represents the time taken to collect the fixed volume of permeate.


(1) $$J\;(\text{L}/{\text{m}}^{2}\text{h})=\Delta V/A\times \Delta t$$

Conductivity of the feed and permeate solutions were measured using a Hanna HI2030-02 conductivity meter (Hanna Instruments, Rhode Island, USA). To convert these conductivity readings into corresponding salt concentrations, a calibration curve was meticulously applied. The salt rejection percentage (*R%*) was subsequently determined using Equation 2, wherein *C_p_* and *C_f_* denote the salt concentration of the filtrate and input solutions, respectively.


(2) $$R\%=(1-C_{p}/C_{f})\times 100$$

Membranes were fabricated in sets of three for each composition, ensuring robustness and reliability in the results. The mean and standard deviation were calculated to understand the data variability.

## 3. Results and discussion

### 3.1. FTIR analysis

**Figure 3:**
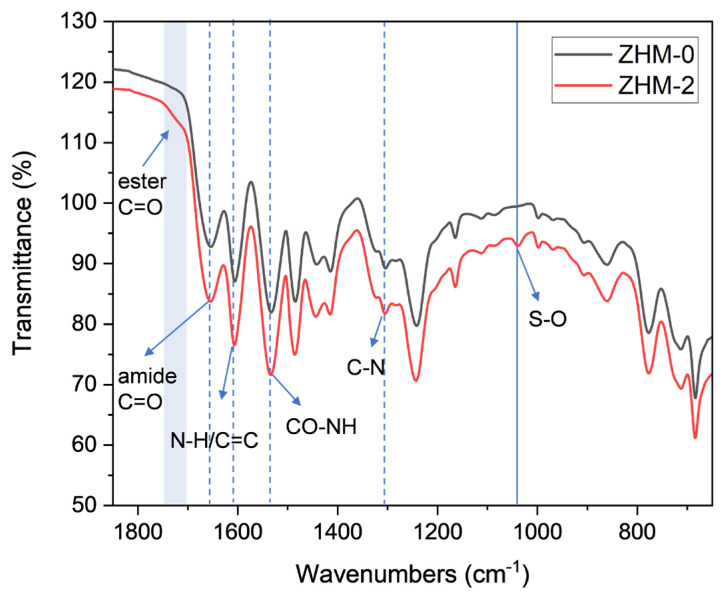
FTIR spectra of the PA active layers of the control membrane (ZHM-0) and ZHM-modified membrane (ZHM-2).

To ascertain the inclusion of zwitterionic sulfobetaine groups within the PA active layer, FTIR spectroscopy analyses were conducted on both the control PA active layer (ZHM-0) and the modified or integrated PA active layer (ZHM-2) produced with 2.0% ZHM in the aqueous phase. The FTIR spectra are depicted in Figure 3. In the FTIR spectrum of the control PA active layer (ZHM-0), characteristic peaks corresponding to amide C=O, N-H/C=C, and CO-NH bonds were observed at 1654, 1607, and 1535 cm^−1^, respectively. In contrast to the spectrum of the control PA active layer (ZHM-0), the modified PA active layer (ZHM-2) had a distinct shoulder peak 1700–1750 cm^−1^, attributed to the C=O group of ester bonds formed between the hydroxyl (−OH) groups of ZHM and the acid chlorides of TMC. Furthermore, a peak at 1039 cm^−1^ was present exclusively in the spectrum of the ZHM-modified PA active layer (ZHM-2), absent in the spectrum of the control PA active layer (ZHM-0). This peak was attributed to the stretching vibration of O=S=O groups, indicating the successful incorporation of zwitterionic sulfobetaine groups into the PA active layer.

### 3.2. WCA analysis

**Figure 4:**
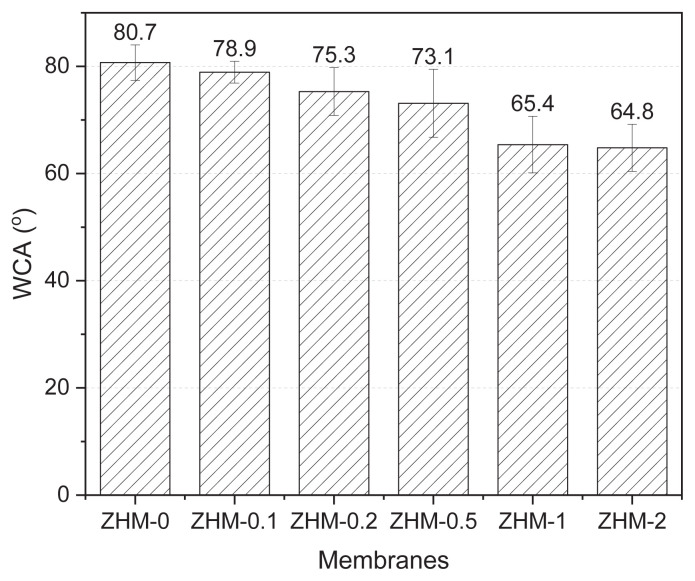
WCAs of the PA top (active) surfaces of the control membrane (ZHM-0) and ZHM-modified membranes (ZHM-0.1, ZHM-0.2, ZHM-0.5, ZHM-1, and ZHM-2).

Zwitterionic structures possess inherent hydrophilicity owing to their electrostatically induced hydration. Consequently, incorporating zwitterionic structures into membrane surfaces is anticipated to enhance their hydrophilic properties. WCA analyses were conducted on the membrane surfaces to assess their hydrophilic characteristics. Figure 4 shows the surface WCAs of the control PA membrane (ZHM-0) and modified PA membranes (ZHM-0.1, ZHM-0.2, ZHM-0.5, ZHM-1, and ZHM-2). ZHM-0 had a surface WCA of 80.7°. As the concentration of ZHM in the modified membranes increased, a reduction in surface contact angles was noted, suggesting enhanced surface hydrophilicity. In the modified membranes, where ZHM was added in concentrations of 0.1%, 0.2%, 0.5%, 1.0%, or 2.0% to the water-based solutions during interfacial polymerization, the surface contact angles decreased progressively. The surface contact angles decreased from 80.7° to 78.9°, 75.3°, 73.1°, 65.4°, and 64.8°, respectively, reflecting the increasing hydrophilic nature of the membrane surfaces with higher ZHM concentrations.

### 3.3. XPS analysis

**Figure 5:**
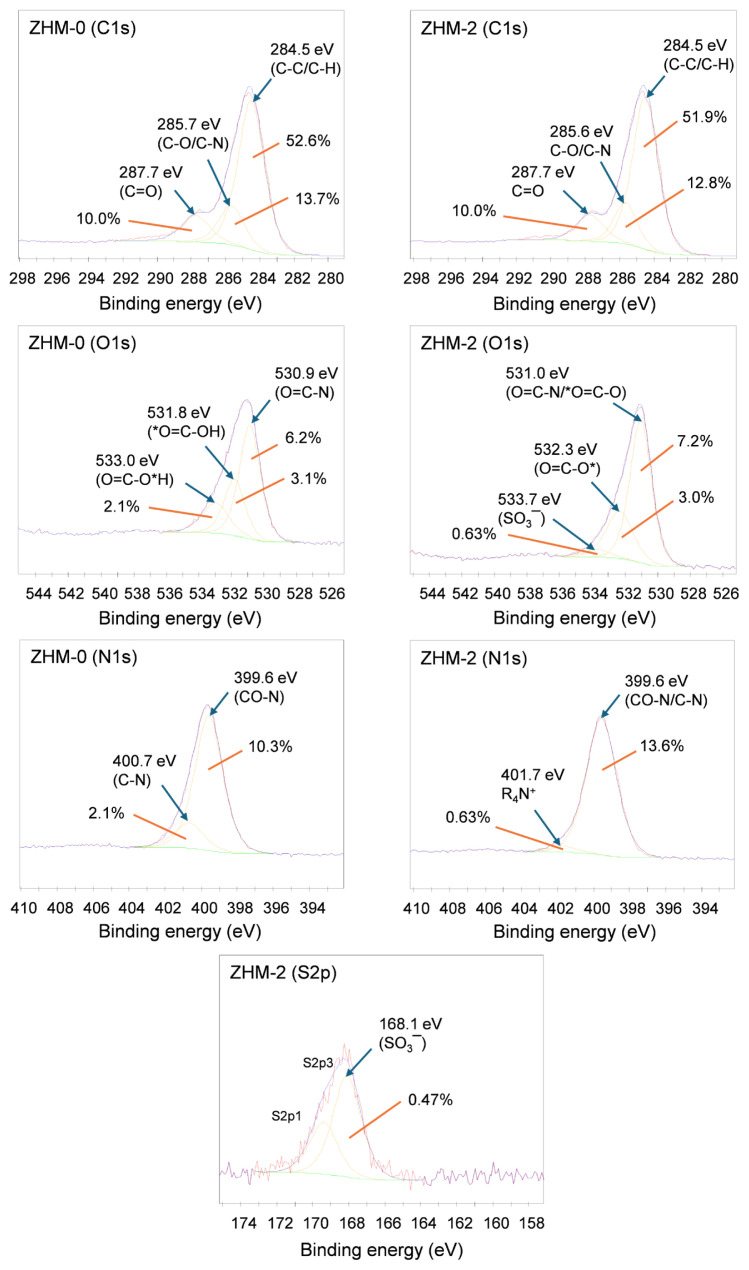
High-definition XPS spectra for C1s, O1s, N1s peaks of the control membrane (ZHM-0) and C1s, O1s, N1s, S2p peaks of the zwitterionic-modified membrane (ZHM-2).

The surfaces of the control membrane (ZHM-0) and zwitterionically modified membrane (ZHM-2) were analyzed by XPS to understand the chemical formula and atomic profile of the membrane surfaces and to prove the embedding of zwitterionic sulfobetaine groups into the PA active layer of the modified membrane. While characteristic peaks matching C1s, O1s, and N1s were observed in both membranes, there was an additional S2p peak in the modified membrane. This shows that the control and modified membrane surfaces had different chemical structures. Figure 5 presents high-definition XPS spectra illustrating the C1s, O1s, and N1s signals of the control membrane (ZHM-0), as well as the C1s, O1s, N1s, and S2p signals of the zwitterionic-modified membrane (ZHM-2). In the high resolution C1s band of the control membrane, 3 signals were identified at bond energies of 284.5, 285.7, and 287.7 eV, corresponding to C-C/C-H, C-O/C-N, and C=O connections, respectively. These peaks were also observed in the zwitterionic-modified membrane (ZHM-2); however, alterations in bond ratios suggested disparities in the structures of the control and zwitterionically modified membranes. The high resolution O1s spectrum of the control membrane showed 3 distinct signals at bond energies of 530.9, 531.8, and 533.0 eV, indicative of amide carbonyl (O=C-N), acid carbonyl (O=C-OH), and acid hydroxyl (O=C-OH) groups, respectively. In the modified membranes, ester groups are generated through the esterification reaction between acid chloride moieties and hydroxyl functionalities of the zwitterionic compound. Due to the incorporation of the additional zwitterionic compound and its esterification reactions, a reduction in acid groups is expected in the modified membranes. The high resolution O1s spectrum of the modified membrane (ZHM-2) had 3 distinctive signals at binding energies of 531.0, 532.3, and 533.7 eV, corresponding to amide or ester carbonyl (O=C=N/O=C-O), ester oxygen (O=C-O), and zwitterionic SO_3_^−^ bonds, respectively. Alterations in bond energies and ratios, alongside the presence of ester and SO_3_^−^ groups, provide evidence for the insertion of zwitterionic functionalities within the PA active layer. In the high resolution N1s spectrum of the control membrane (ZHM-0), 2 distinct peaks were identified at binding energies of 399.6 and 400.7 eV, associated with amide (O=C-N) and C-N bonds, respectively. A new peak emerged in the high-definition N1s spectrum of the modified membrane (ZHM-2) at 401.7 eV, attributed to the quaternary ammonium group present in the zwitterionic-modified membrane. Likewise, the emergence of the S2p signal at a binding energy of 168.1 eV in the zwitterionic-modified membrane, absent in the control membrane, was ascribed to the existence of the SO_3_^−^ group inherent in the zwitterionic structure. Collectively, these findings provide conclusive proof of successful zwitterionic modification of the PA active layer.

### 3.4. FESEM analysis

**Figure 6:**
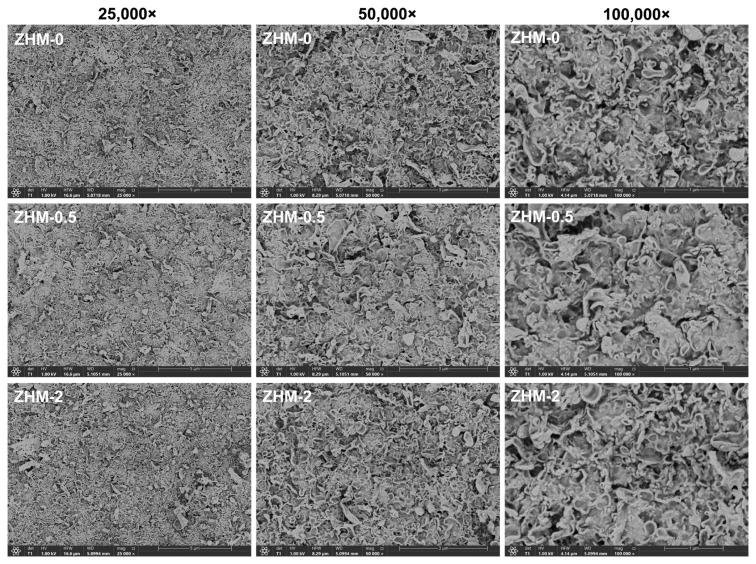
FESEM active surface configurations of the control PA membrane (ZHM-0) and ZHM-integrated modified PA membranes (ZHM-0.5 and ZHM-2).

The surface morphological traits of the membranes were inspected using FESEM. Figure 6 illustrates FESEM images depicting the active surfaces of both the control membrane (ZHM-0) and the ZHM-integrated modified membranes at magnifications of 25,000×, 50,000×, and 100,000×. The characteristic rugged top surface morphology mirroring a ridge-and-valley appearance—a typical feature of the active surface layer in aromatic PA RO membranes—was evident across all membranes, slight alterations in the morphological surface structures were observed with the addition of ZHM. Ear-like protrusions were also noted on the surfaces of PA membranes modified by ZHM. However, it was evident that the jagged structure and ridge-and-valley appearance diminished with the inclusion of ZHM. The ridge-and-valley appearance observed in the control membrane can be attributed to the inflexible aromatic structures. In the modified membranes, these structures diminish and loosen with higher ZHM concentrations used during membrane fabrication. The introduction of more flexible aliphatic zwitterionic groups filling the gaps between aromatic groups may contribute to the reduction in ridge appearance.

### 3.5. Desalination performance

The flux and salt retention performance of the membranes were evaluated by filtering 2000 ppm NaCl or MgSO_4_ aqueous salt solutions through them in a conventional dead-end pressure filtration system equipped with a magnetic stirrer. Filtrations were conducted at room temperature, applying a pressure of 15 bar, and maintaining a rotation speed of 300 rpm for the feed solution.

#### 3.5.1. NaCl solution filtration

**Figure 7:**
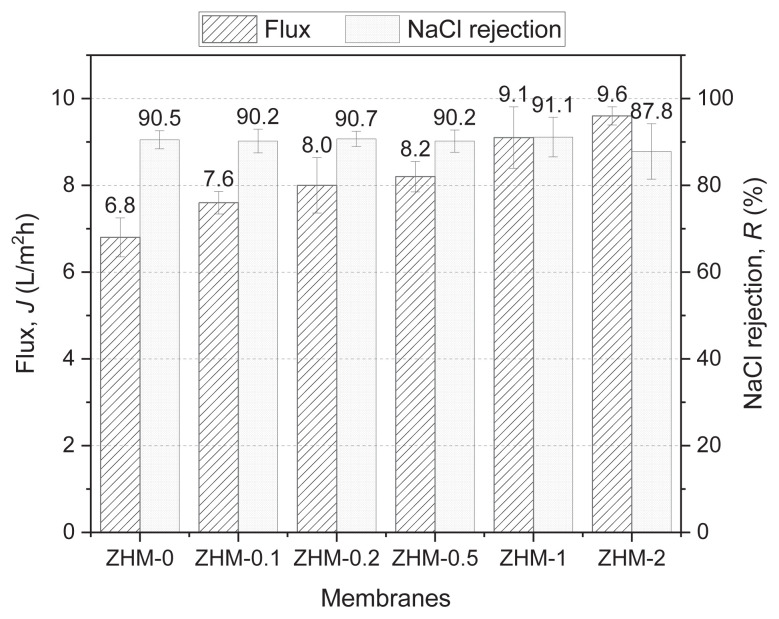
Desalination performance of the control membrane (ZHM-0) and the modified membranes (ZHM-0.1, ZHM-0.2, ZHM-0.5, ZHM-1, and ZHM-2) after filtering a 2000 ppm NaCl solution.

The flux and salt retention values obtained from the NaCl filtration experiments are depicted in Figure 7. In the filtration of a 2000 ppm aqueous NaCl feed solution, the control membrane (ZHM-0) without ZHM had a flux of 6.8 L/m^2^h and a salt retention of 90.5%. The flux noticeably improved in the ZHM-integrated modified membranes, owing to the addition of highly hydrophilic zwitterionic structures within and on the PA active layer [35,47]. Zwitterionic structures offer antifouling properties and enhanced wettability by augmenting hydrophilic surface properties driven by electrostatic hydration. They can facilitate water transmission by establishing electrostatic water-attracting routes or channels within the membrane structure [18,32]. The fluxes of ZHM-0.1, ZHM-0.2, ZHM-0.5, ZHM-1, and ZHM-2 membranes improved to 7.6, 8.0, 8.2, 9.1, and 9.6 L/m^2^h, respectively, without any observable decrease in their salt rejection values. Typically, there is an inverse relationship between salt retention and flux, making it challenging to increase both parameters simultaneously without sacrificing one over the other. Relative to the control membrane (ZHM-0), the salt retention remained the same while the flux increased from 6.8 to 9.1 L/m^2^h in the modified membrane ZHM-1. The integration of zwitterionic, hydrophilic, and flexible aliphatic structures into the PA matrix from a singular point, without substantial deviation from the primary structure, led to an increase in membrane flux and provided selective properties to the membrane. Besides imparting hydrophilic characteristics, zwitterionic structures may induce ionic cross-linking and electrostatic ionic repulsions, thereby enhancing NaCl retention. In the modified PA membranes ZHM-0.1, ZHM-0.2, ZHM-0.5, ZHM-1, and ZHM-2, the salt rejection values were 90.2%, 90.7%, 90.2%, 91.1%, and 90.1%, respectively.

#### 3.5.2. MgSO_4_ solution filtration

**Figure 8:**
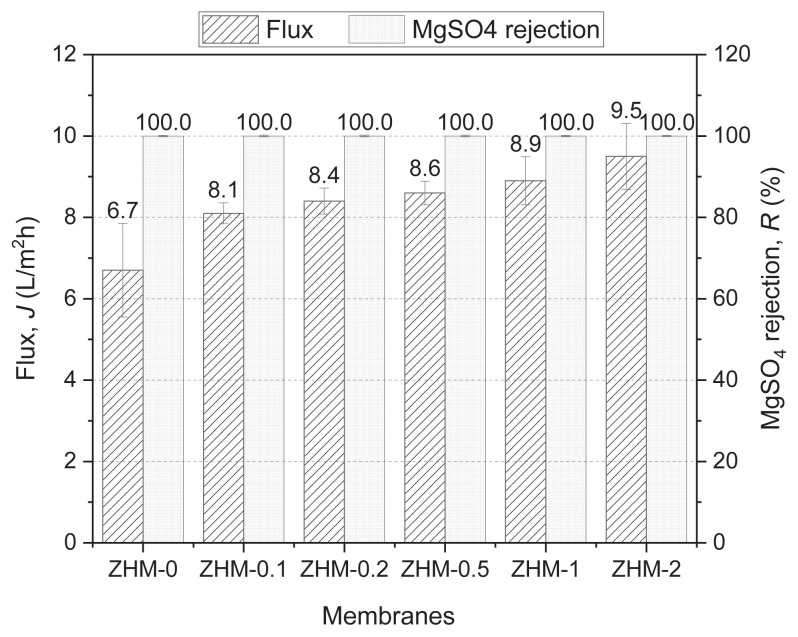
Desalination performance of the control membrane (ZHM-0) and the modified membranes (ZHM-0.1, ZHM-0.2, ZHM-0.5, ZHM-1, and ZHM-2) after filtering a 2000 ppm MgSO_4_ solution.

Figure 8 presents the flux and salt retention results of the MgSO_4_ filtration experiments. When filtering a 2000 ppm aqueous MgSO_4_ feed solution, the control PA membrane (ZHM-0) had a flux of 6.7 L/m^2^h and a salt retention of 100%. Upon integrating ZHM into modified membranes, flux values increased with increasing ZHM concentration, while salt rejections remained consistent at 100%. Comparative analysis with the control membrane showed noteworthy improvements in flux across ZHM-0.1, ZHM-0.2, ZHM-0.5, ZHM-1, and ZHM-2. Specifically, the fluxes observed were 8.1, 8.4, 8.2, 8.6, 8.9, and 9.5 L/m^2^h, respectively. This means that the flux substantially increased by up to 41.8% without any loss of salt retention.

For the first time, the synthesized ZHM was used as an additive in the modification of the PA active layer formed by interfacial polymerization of TMC and MPD monomers. The ZHM additive is a highly promising candidate for PA-TFC desalination membrane applications due to its easy synthesis, straightforward applicability in PA-TFC production, and its significant impact on separation performance. Zwitterionic structures can form electrostatically induced hydrophilic channels within the membrane matrix, promoting efficient water transport [35,47,50]. These structures can maintain selectivity through ionic interactions while also enhancing water permeability. Additionally, zwitterionic surfaces resist fouling from biomolecules and other contaminants due to their inherent hydrophilicity, which prevents the adhesion of foulants and supports long-term membrane performance [29,51]. The versatility of zwitterionic structures in offering both high permeability and selectivity, along with fouling resistance, makes them an attractive option for improving membrane performance. Zwitterionic structures have been incorporated into membranes using various methods, including grafting, deposition, and coating on the membrane surface, as well as by integrating them directly into the membrane matrix through blending or chemical reactions [17,18,20,23]. While surface modifications for zwitterionic functionalization often involve more complex and time-consuming methods, incorporating zwitterionic monomers via interfacial polymerization is a simpler and more practical approach, provided that the monomer synthesis itself is not inherently difficult or time consuming [43,52,53]. In literature, only a limited number of zwitterionic RO membranes have been developed through the incorporation of zwitterionic monomers via interfacial polymerization [34,36,47]. For example, AEPPS was incorporated into an aqueous MPD solution, where it reacted with TMC through interfacial polymerization to fabricate TFCMZs [47]. The introduction of AEPPS significantly improved the surface hydrophilicity and antifouling properties of the membrane. Under brackish water desalination conditions (2000 ppm NaCl feed solution, 1.5 MPa, 25 °C), the water flux of TFCMZs containing 10% AEPPS reached 54.5 ± 3.2 L/m^2^h, representing an approximately 82% enhancement compared to the pristine membrane, while maintaining NaCl rejection above 98%. Table 2 presents a comparison of the separation performance from this study with other research on zwitterionic modifications of RO/NF membranes available in the literature. Table 2 highlights the variation in membrane preparation techniques and filtration parameters across different studies, including differences in their feed solution composition, concentration, filtration pressure, and system design. These discrepancies make direct comparisons challenging. For instance, even when using the same membrane, flux values can vary significantly depending on whether a dead-end or crossflow filtration system is used. Therefore, it is more accurate to compare the percentage changes in flux or salt retention in modified membranes in relation to their respective controls. This approach provides a clearer understanding of the enhancements brought about by the modifications. Among the studies listed in Table 2, the current study demonstrates a substantial improvement in flux, showing a 34%–42% increase compared to its control, while maintaining consistent salt rejection performance at 91% for NaCl and 100% for MgSO_4_. In RO membranes, achieving a high flux while maintaining high NaCl retention is a significant challenge, as evidenced by this study. In a number of other zwitterionic RO membrane studies listed in Table 2, salt retention in modified membranes remains almost the same as in the control membrane and the percentage improvement in flux is lower compared to this study [28, 34, 54–56]. Li et al. [57] and Weng et al. [58] (Table 2) fabricated zwitterionic NFMs via interfacial polymerization of zwitterionic monomers with TMC. Although these membranes have high flux values or flux change rates, their NaCl salt retention is considerably lower (23.5% and 14.3%, respectively) compared to the current study.

## 4. Conclusions

This research introduces a novel, easy applicable, and performance-effective approach aimed at modifying the aromatic PA active layer in PA-TFC RO desalination membranes by utilizing a ZHM. ZHM synthesis involved the interaction of N,N-dimethylpropanolamine with 1,3-propane sultone. ZHM was incorporated into the PA active layer by interfacial polymerization. During this process, ZHM was introduced into the aqueous phase alongside MPD, while TMC was incorporated into the hexane phase. Esterification reactions between the hydroxyl (−OH) group of ZHM and the acid chloride group of TMC incorporated ZHM into the PA active layer. Different zwitterionic-modified PA active layered TFC membranes were prepared by adding varying amounts (0.1, 0.2, 0.5, 1, and 2% w/w) of ZHM to the aqueous solutions. The performance efficiency of the prepared zwitterionic-modified PA membranes was compared to that of the control PA membrane prepared without ZHM. The manufactured membranes were chemically and morphologically characterized to confirm the embedding of ZHM into the PA active layer. FTIR and XPS analyses of the resulting membranes showed that zwitterionic structures were efficiently incorporated into the PA active layers in the modified membranes. Salt retention and water flux often vary often, making it challenging to improve both parameters simultaneously without compromising one. The modified membranes in this study enhanced flux performance without sacrificing salt rejection. Due to the monohydroxyl functionality of ZHM, hydrophilic properties were imparted to the membrane without deviating from its basic structure. This was achieved by integrating zwitterionic structures into the primary aromatic framework, without forming a bridge between the aromatic groups of the PA active layer. Within the membrane framework, zwitterionic functionalities boosted water permeability by generating electrostatic hydrophilic routes/channels. Additionally, they facilitated salt rejection by ionic crosslinking and electrostatic repulsions. The desalination capability of all membranes was assessed using a dead-end flow filtration unit. This filtration involved passing a 2000 ppm NaCl or MgSO_4_ saltwater solution through the membranes at room temperature, using an operating pressure of 15 bar. The incorporation of ZHM led to an increase in flux values in the modified membranes without adversely impacting their salt rejection values in comparison to the control membrane. For example, during filtration of the MgSO_4_ aqueous solution, the control membrane had a flux value of 6.7 L/m^2^h with 100% salt rejection. With the addition of ZHM, flux values increased up to 9.5 L/m^2^h, while salt rejections consistently remained at 100%.

## Supplementary information

Figure S1 ^1^H NMR spectra of the zwitterionic hydroxyl compound ZHM (500 MHz, CD_3_OD).

Figure S2 ^13^C NMR spectra of the zwitterionic hydroxyl compound ZHM (500 MHz, CD_3_OD).

## Figures and Tables

**Table 1:** Weight percentages of components of water and hexane phases used in interfacial polymerizations.

Membranes	Water phase					ZHM/MPD weight ratio	Hexane phase
	
	ZHM (wt.%)	MPD (wt.%)	TEA (wt.%)	CSA (wt.%)	SDS (wt.%)		TMC (wt.%)
ZHM-0	0.0	2.0	1.0	1.0	0.1	0.00	0.1
ZHM-0.1	0.1	2.0	1.0	1.0	0.1	0.05	0.1
ZHM-0.2	0.2	2.0	1.0	1.0	0.1	0.10	0.1
ZHM-0.5	0.5	2.0	1.0	1.0	0.1	0.25	0.1
ZHM-1	1.0	2.0	1.0	1.0	0.1	0.50	0.1
ZHM-2	2.0	2.0	1.0	1.0	0.1	1.00	0.1

**Table 2:** Comparison of the separation performance of zwitterionic RO/NF membranes in studies reported in the literature^a^ and the current study.

Membrane Support	Zwitterion modification	Monomer preparation/Modification method b	Filtration test conditions c	Flux (L/m^2^h) d	Flux Change (%)	Rejection (%) d	Salt Rejection Change (%)	Ref.
PSF	ZHM (0.1, 0.2, 0.5, 1.0, or 2.0 wt.%) as a monomer additive	Chemical reaction/IP	-2000 ppm NaCl or MgSO_4_ -Dead-end filtration system -15 bar -rt	NaCl: 9.1 [6.8] MgSO_4_: 9.5 [6.7]	+33.8% +41.8%	NaCl: 91.1 [90.5] MgSO_4_: nc^e^ [100]	+0.7% nc	This study
PSF	PVA-arginine	Esterification reaction/ “grafting-to”	-2000 ppm NaCl -Cross-flow filtration system -16 bar -rt	NaCl: 57.2 [52.8]	+8.3%	NaCl: 99.5 [95.6]	+4.1%	[54]
PSF	L-lysine	-/blending + IP	-2000 ppm NaCl -Cross-flow filtration system -16 bar -20 °C	NaCl: 58.2 [49.2]	+18%	NaCl: 98.4 [98.2]	+0.2%	[55]
Commercial RO	PSBMA + MTAC	-/ARGET-ATRP	-2925 ppm NaCl -Cross-flow filtration system -24.1 bar -rt	NaCl: 62.7 [65.1]	−3.7%	NaCl: 97.9 [98.2]	−0.3%	[56]
Commercial RO	MPPS	Chemical reaction / solution coating	-2000 ppm NaCl -Dead-end filtration system -20 bar -rt	NaCl: 13.3 [16.2]	−17.9%	NaCl: 97.1 [96.4]	+0.7%	[28]
PSF	SPPT	Chemical reaction/IP	-32000 ppm NaCl -Cross-flow filtration system -55 bar -rt	NaCl: 33 [25]	+32%	NaCl: 98.9 [98.8]	+0.1%	[34]
PSF	PPD-MEPS	Chemical reactions/ blending+IP	-500 ppm Na_2_SO_4_/NaCl -Cross-flow filtration system -5 bar -rt	Na_2_SO_4_/NaCl: 49 [34]	+45%	Na_2_SO_4_: nc [95] NaCl: 23.5 [38.3]	nc −38.6%	[57]
hPAN	AEPPS	Chemical reaction/IP	-1000 ppm NaCl or Na_2_SO_4_ -Cross-flow filtration system -6 bar -25 °C	Water: 80.3 [−] (water filtration)	Na^f^	NaCl: 14.3 [−] Na_2_SO_4_: 78.1 [−]	Na Na	[58]

a All values represent the best performance achieved under the optimal modification conditions.

b IP: interfacial polymerization.

c rt: room temperature (~25 °C).

d Zwitterionic-modified membrane [control membrane].

e nc: no change.

f Na: not available

## Footnotes

1 Cadotte JE (1981). Interfacially synthesized reverse osmosis membrane. Google Patents [online]. Website https://patents.google.com/patent/US4277344A/en [accessed 22 January 2024].
