# Supplementary figures and images for: Optimizing polyamide thin-film composite desalination membranes: simple and performance-effective modification by zwitterionic monohydroxyl monomer
Source: Turk J Chem. 2025 Apr 25;49(4):404–18. doi: 10.55730/1300-0527.3740 (PMC12425390; doi:10.55730/1300-0527.3740)

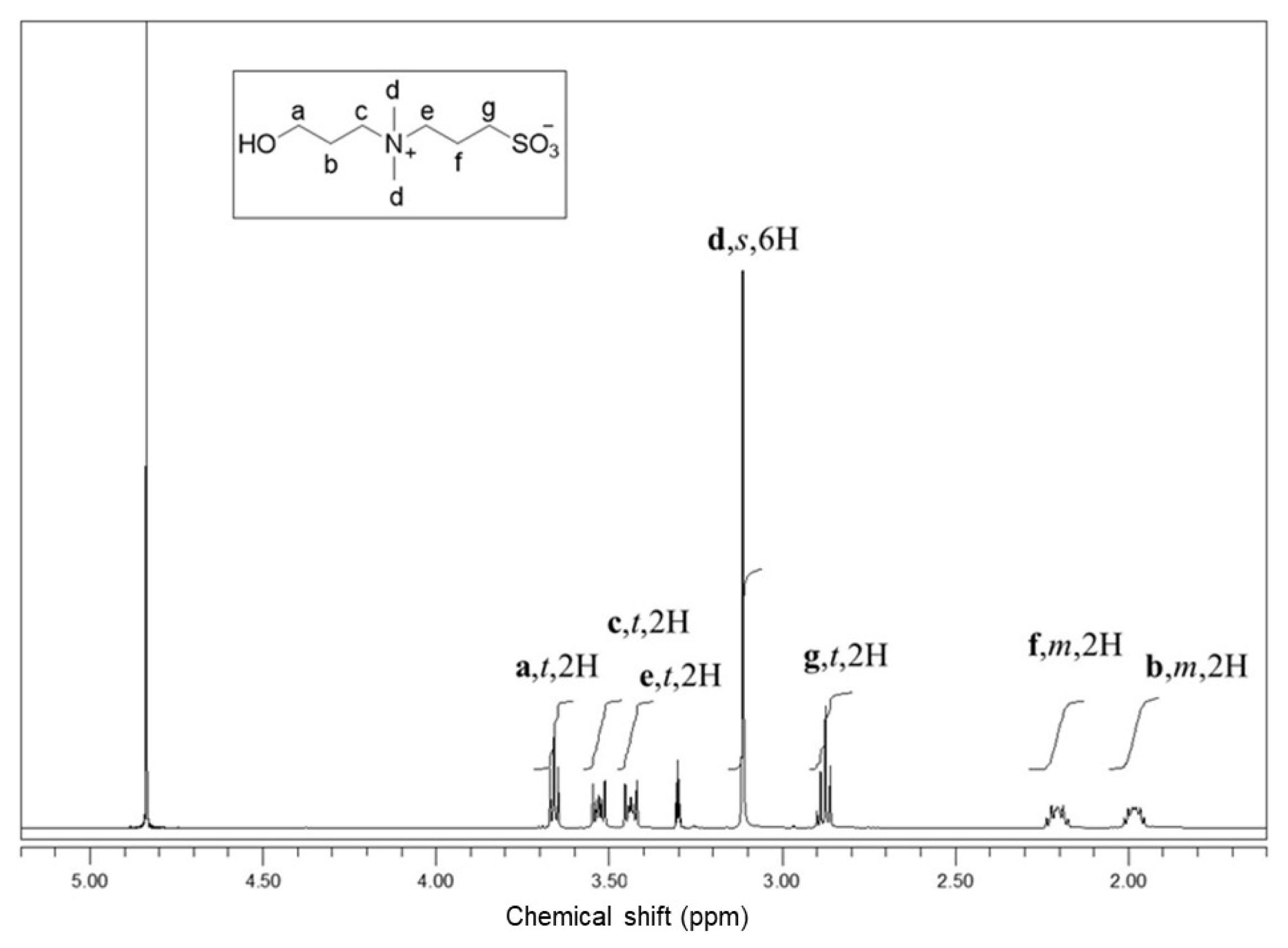

Supplement: Figure S1 — 1H NMR spectra of the zwitterionic hydroxyl compound ZHM (500 MHz, CD3OD). [file tjc-49-04-404s1.tif]

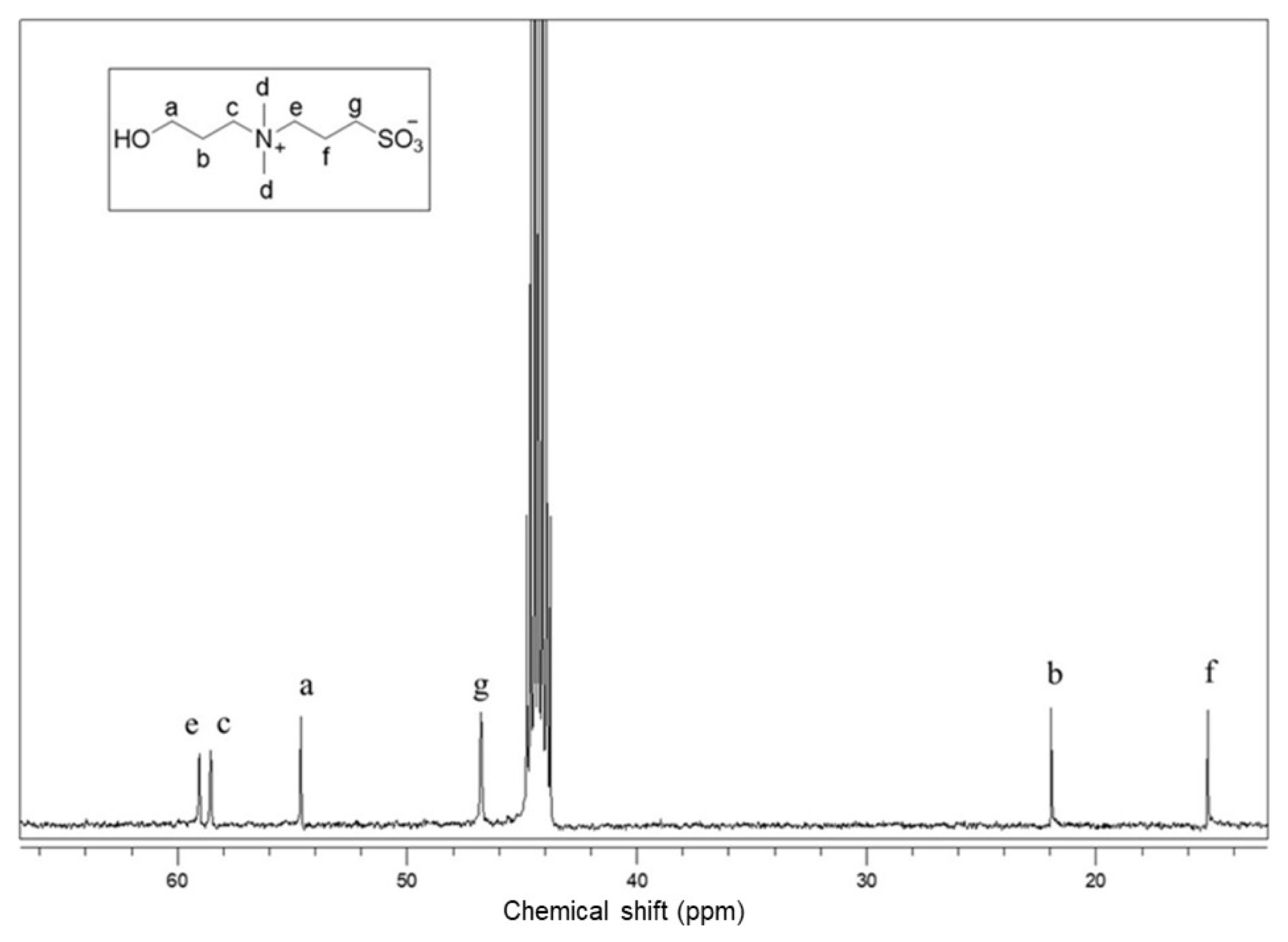

Supplement: Figure S2 — 13C NMR spectra of the zwitterionic hydroxyl compound ZHM (500 MHz, CD3OD). [file tjc-49-04-404s2.tif]
